# Consequences of Misspecifying Levels of Variance in Cross-Classified Longitudinal Data Structures

**DOI:** 10.3389/fpsyg.2016.00695

**Published:** 2016-05-18

**Authors:** Jennifer Gilbert, Yaacov Petscher, Donald L. Compton, Chris Schatschneider

**Affiliations:** ^1^Department of Special Education, Vanderbilt UniversityNashville, USA; ^2^Florida Center for Reading Research, Florida State UniversityTallahassee, USA; ^3^Department of Psychology, Florida State UniversityTallahassee, USA

**Keywords:** cross-classified model, oral reading fluency, longitudinal analysis, multilevel modeling, reading

## Abstract

The purpose of this study was to determine if modeling school and classroom effects was necessary in estimating passage reading growth across elementary grades. Longitudinal data from 8367 students in 2989 classrooms in 202 *Reading First* schools were used in this study and were obtained from the Progress Monitoring and Reporting Network maintained by the Florida Center for Reading Research. Oral reading fluency (ORF) was assessed four times per school year. Five growth models with varying levels of data (student, classroom, and school) were estimated in order to determine which structures were necessary to correctly partition variance and accurately estimate standard errors for growth parameters. Because the results illustrate that not modeling higher-level clustering inflated lower-level variance estimates and in some cases led to biased standard errors, the authors recommend the practice of including classroom cross-classification and school nesting when predicting longitudinal student outcomes.

## Introduction

Students are educated in complex environments consisting of curricula, teachers, peers, schools and beyond, all of which influence growth in achievement. Researchers have conducted many studies investigating how features of educational contexts affect student achievement. For example, class size (Blatchford et al., 2007), concentration of students with problem behaviors (Koth et al., 2008), type of instruction (Silverman, 2007), teacher quality (Borman and Kimball, 2005), school climate (Johnson and Stevens, 2006), and school composition (Goldsmith, 2004) have all been investigated as potential predictors of student outcomes. Research on context effects acknowledges that students who share an environment are exposed to similar influences that lead them to be more similar to one another than to students outside their immediate context (Raudenbush and Bryk, 2002). Students' outcomes, therefore, are dependent not only on their own personal characteristics but also the characteristics of their surroundings. Multilevel models have been developed to account the lack of independence of observation that arises from the fact that students (or individuals) who share an environment are exposed to similar influences that lead them to be more similar to one another than to students outside their immediate context (Raudenbush and Bryk, 2002).

The effect of shared contexts (also known as statistical dependency) can be quantified by the intraclass correlation (ICC), or the proportion of total variance lying between units at a given level. Hedges and Hedberg (2007) used nationally representative databases to calculate average ICCs between schools. In terms of student reading achievement across grades K-12, the average ICC was 0.22 across all schools, 0.19 in low-socioeconomic status schools, and 0.09 in low-achievement schools. From their synthesis, it appears that between 9 and 22% of the variance in student reading achievement lies between schools. In other words, a considerable proportion of the differences in student reading achievement can be explained by differences in the schools that the students attend. Classroom ICCs have not been estimated with a representative sample of classrooms, yet a study on the unidimensionality of literacy across grades one through four by Mehta et al. (2005) provides rough estimates. The authors found that the average classroom ICC in word reading, passage comprehension, and phonological awareness was 0.15, 0.17, and 0.22, respectively. These estimates represent substantial classroom influences on students' performance in the domain of reading, although they might be somewhat biased because school variance was not taken into account. Nevertheless, the ICCs reported for schools and classrooms are evidence that classroom and school membership is necessary to consider when analyzing student reading achievement.

### Ignoring contexts

Ignoring the contexts in which students are educated is problematic for both theoretical and statistical reasons. Theoretically, it gives an incomplete picture of the sources of influences that affect student performance. When effects of classrooms and schools are ignored, the variability in student performance is assumed to be associated only with students' own characteristics rather than considering variability in classroom and school environments. This limits the types of research questions that can be explored to only those involving student-level predictors, and it misrepresents the statistical impact of those predictors (due to biased standard errors) because other sources of variation have been ignored. When interventions are implemented at the classroom or school level, it is particularly important that researchers use models that account for clustering effects at the classroom or school level. Otherwise, teacher, tutor, group dynamics, or other contextual factors could obscure the observed treatment effects.

Statistically, ignoring contexts by conducting a subject-level analysis when dependency in the data exists violates the assumption of independence required to use the *t*- and *F*-distributions needed for statistical testing. The result is inflated variance at the level of the subject (Meyers and Beretvas, 2006), standard errors with a downward bias, and thus inflated likelihood of Type I errors (Raudenbush and Bryk, 2002). These problems are similar to those associated with omitted variable bias, a phenomenon most widely discussed in regard to regular regression analyses (Barreto and Howland, 2006). Therefore, the best method for handling data with nested data is the use of multilevel models which simultaneously incorporate multiple levels of influence and provide efficient estimates from which researchers can make valid inferences (Hox, 2002; Raudenbush and Bryk, 2002; Goldstein, 2003). Raudenbush and Bryk (2002) report that many studies have failed to use multilevel models despite the hierarchical structures inherent in their data.

### Data structures

When lower-level units are embedded within a hierarchy where they belong to one and only one unit at each higher level (e.g., students in classrooms and classrooms in schools), there exists a (hierarchically) nested structure (Goldstein, 1986). Nesting also occurs when assessments are obtained over multiple observations on the same individual (observations nested within individuals). Raudenbush and Bryk (2002) regard education as the leading example of nested structures because repeated assessments are nested within students and students are nested in classes and classes are nested in schools and schools are nested in districts.

Not all data with dependency are nested, however. When lower-level units are members of more than one higher-level unit, they are considered cross-classified in those higher-level contexts^1^ (Goldstein, 1987). Cross-classification can occur both within- and across-years. Within a given year, students may be members of several contexts. In elementary school, for example, students who receive small-group reading intervention are often recruited from different classrooms. In this situation, students are cross-classified by small-groups and classrooms, not hierarchically nested within them. Similarly, in grades during the high-school years, students are members of several content-related classrooms such that each classroom contains a different make-up of students; in this case, students are cross-classified by classrooms. In across-year longitudinal data, cross-classification can occur due to students' changing environments (e.g., classrooms) over time. For example, students from one kindergarten classroom disperse into several first-grade classrooms and then into several second-grade classrooms. Because students are affected by their surrounding contexts within and across time, one should not ignore the nested and/or cross-classified structure when conducting statistical analyses (Raudenbush and Bryk, 2002).

### Ignoring levels

#### Nested data

The necessity of accounting for dependency in data is well-established, and the consequences for failing to do so have been conveyed in studies using both simulated and empirical data. For hierarchically nested simulated data, Moerbeek et al. (2003) found that ignoring the highest level of nesting in a 2-level model produced incorrect standard errors and confidence intervals for the fixed effects. In a 3-level model, the consequence of ignoring the uppermost level was inflated variance estimates of the intermediate level, but variance of the lowest level remained unchanged (Moerbeek, 2004). These results only held true for balanced designs, however. For unbalanced designs, standard errors at all levels were incorrect and the fixed and variance estimates were also biased. Hutchison and Healy (2001), Opdenakker and Van Damme (2000), and Tranmer and Steele (2001) found similar results using real data with three levels. Misattributed variance and biased standard errors serve as evidence against ignoring levels when estimating models with hierarchically nested data.

#### Cross-classified data

It appears that cross-classified data are subject to consequences similar to nested data. Meyers and Beretvas (2006) used two models to explore the effects of ignoring cross-classified data structures. One model accounted for the cross-classification of students in middle and high schools and the other ignored students' middle school membership and only accounted for students' nesting within high schools. Results showed that fixed effects across models were similar, but the variance components diverged such that the variance attributed to the remaining crossed factor (τ = 15.98) in the nested model was overestimated when compared to the cross-classified model (τ = 8.37) which included both crossed factors. Meyers and Beretvas concluded that estimated standard errors will become increasingly negatively biased as variance that is associated with the ignored factor increases.

Luo and Kwok (2009) used real and simulated data to examine the effects of ignoring a level of cross-classification as the nature of the cross-classification differed. In data with balanced cross-classification, the effects of ignoring a crossed factor were basically the same as ignoring a nested factor. When factors were crossed in an unbalanced way, the results differed depending on which crossed factor was ignored, which was partially dependent on the amount of variance associated with that factor. Similar to the results for unbalanced nested models, fixed effects and their standard errors were affected when the crossed factor was ignored. Moreover, Fielding (2002) found that accounting for the cross-classification lead to more precise residuals and standard errors than when cross-classification was ignored.

#### Longitudinal data

The above studies demonstrate the effects of ignoring levels of data for static, single-outcome models. Because the inclusion of higher levels is important in those contexts, it is reasonable to assume growth models may be affected in similar ways. Raudenbush and Bryk (2002) provide statistical evidence in support of taking clustering into account when modeling growth. In their discussion of cross-classified models, they used a subsample of data from the Immersion study conducted by Ramirez et al. (1991) to compare two growth models, one ignoring and one accounting for classroom cross-classification. Because this study spanned kindergarten through sixth grade, each year students were members of classrooms that contained a different set of classmates. Results confirmed that ignoring higher levels of clustered data compromises the integrity of the growth modeling prediction results by producing biased standard errors. Specifically, Raudenbush and Bryk found that the standard errors for the intercept and slope in the two-level model were 2.51 and 2.22, respectively, while they were 4.00 and 3.53, respectively, in the cross-classified model. After estimating the cross-classified model, they concluded that “part of the variability that had been attributed to individual differences [was] now attributed to classroom experience” (p. 393). Thus, just as in the static outcome models, the fixed and random effects in their initial two-level, time-within-student model were subject to Type I error due to the incorrect partitioning of variance.

### Extending the literature

This study extends the literature by providing information about which levels of data are necessary for correctly partitioning variance in cross-year, cross-classified growth models. It extends the work by Raudenbush and Bryk (2002) on cross-classified longitudinal models by adding the school level and providing more model comparisons. This study will serve as practical example of the implications of misspecifying models and how researchers should proceed when making decisions about models that involve similar data structures. We believe this to be a relevant contribution to the field of education as tracking student growth across time has become commonplace with the implementation of both No Child Left Behind (NCLB; 2002)^2^ and responsiveness to intervention (RTI). Our main research question was: What are the consequences of omitting various levels of data represented at the classroom and school levels in cross-year, cross-classified oral reading fluency (ORF) data? We will examine the extent to which fixed effects, their standard errors, and random effects were biased in the various models to answer our question. It should be noted that our objective was only to partition, not explain, variance, which is why no classroom or school covariates were included in the models.

The dependent measure in this study was ORF. As mentioned previously, the current educational system places great emphasis on students' academic growth because of NCLB and RTI, and ORF allows for the monitoring and quantification of growth. Furthermore, ORF is frequently used for making data-based decisions about which students are struggling to acquire basic reading skills and how to proceed with classroom instruction (Reschly et al., 2009). Also, it has been reported that 43 of the United States indicated that they would use ORF measures as part of the *Reading First* program (U.S. Department of Education, 2007). The predictive validity of ORF has been demonstrated by its relation with overall reading as rated by classroom teachers (*r*s range from 0.56 to 0.77 in grades 1–5, Tindal and Marston, 1996), future standardized reading measures (rs range from 0.34 to 0.82 in grades K-3 predicting 3rd grade performance, (Good et al., 2001), high-stakes assessment levels (91% of students reading at or above benchmark earned an adequate level of achievement on the state test in grade 3; (Buck and Torgesen, 2006), comprehension (*r* = 0.57 in grade 3, Spear-Swerling, 2006; 0.67, Riedel, 2007), reading proficiency (*r*s ranged from 0.67 to 0.82 in grades 1–3, Baker et al., 2008), and later reading success (*p* < 0.001 for grade 1 ORF growth predicting grade 3 standardized reading test; Chard et al., 2008). Researchers have used growth in ORF for a variety of purposes including describing early reading development (Fuchs et al., 2004), assessing response to intervention (Linan-Thompson et al., 2007), selecting students for early reading intervention (Compton et al., 2006), and evaluating the accuracy of teacher judgments about the progress of poor readers (Graney, 2008). Not only has ORF been used for a broad range of purposes but it has also been used to assess a broad range of students including those with speech/language impairments (Puranik et al., 2008), reading disabilities (Olinghouse et al., 2006), intellectual disability and emotional/behavioral disorders (Faykus and McCurdy, 1998; O'Connor et al., 1998), academic at-risk designation (Simmons et al., 2008), English-language learner designation (Santoro et al., 2006; Fitzgerald et al., 2008), and Latino students (Al Otaiba et al., 2009). Yet despite the literature using ORF outcomes to understand individual differences in how students change over time, virtually no studies account for students changing classrooms and/or schools over multiple years. With studies such as Luo and Kwok (2009) and Meyers and Beretvas (2006) noting the importance of capturing this data structure, it is important for educational research to understand the impact of not accounting for cross-classification in multilevel models.

## Methods

### Participants

Data used in this study were obtained from an archival data source maintained by the Florida Center for Reading Research. The archival data source maintains data from *Reading First* and non-*Reading First* schools in Florida who reported reading data and received reports of the data. Progress-monitoring in ORF was assessed in first through third grades. Because ORF produced skewed distributions in first grade (Catts et al., 2009), we limited our dependent measure to only waves of data collected in second and third grades. Complete second and third grade data in the archive was comprised of 9835 students in 985 second grade classrooms and 1025 third grade classrooms in 202 *Reading First* schools throughout the state of Florida. Students were cross-classified by classrooms in each grade because classroom membership varied as students advanced from second to third grade.

Furthermore, for model specification purposes, the sample was limited to students who remained in the same classroom within a grade and remained in the same school for both grades 2 and 3. Nearly 15% of the sample was excluded for changing classrooms or schools or leaving the study completely, which left 8367 students in the remaining sample. Those students belonged to 147 schools (total students per school varied between 10 and 106), 924 different second grade classrooms (total students per class varied from 1 to 23), and 937 different third grade classrooms (total students per class varied from 1 to 25). Table 1 provides descriptive information about the remaining sample. In general, almost half the sample was male and half was of Caucasian descent. The majority of the sample received free or reduced lunch prices, and almost a fifth had some type of special education label. Correlations among waves of ORF in the sample are presented in Table 2.

**Table 1:** **Descriptive information (*N* = 8367)**.

	***f***	***%***
Free/reduced price lunch	5474	65.42
Male	4217	50.40
Race		
African Am.	1854	22.16
Asian	151	1.80
Hispanic	2230	26.65
Multiracial	357	4.27
Caucasian	3754	44.87
Other ethnicity	21	0.25
Special education label	1898	22.68

**Table 2:** **Correlations among waves of oral reading fluency**.

**Measure**	**1**	**2**	**3**	**4**	**5**	**6**	**7**	**8**
1. ORF, Sept. 2nd grade	–							
2. ORF, Dec. 2nd grade	0.93	–						
3. ORF, Feb. 2nd grade	0.91	0.91	–					
4. ORF, Apr. 2nd grade	0.88	0.89	0.93	–				
5. ORF, Sept. 3rd grade	0.89	0.89	0.92	0.92	–			
6. ORF, Dec. 3rd grade	0.86	0.88	0.90	0.90	0.93	–		
7. ORF, Feb. 3rd grade	0.82	0.83	0.88	0.89	0.90	0.91	–	
8. ORF, Apr. 3rd grade	0.82	0.83	0.87	0.88	0.90	0.91	0.92	–
Mean	65.79	65.25	82.15	97.28	80.13	92.99	103.18	108.53
SD	31.77	31.69	32.94	33.51	33.13	33.81	32.85	33.74

*ORF = oral reading fluency (words per minute). All correlations significant at p < 0.0001. n's ranged from 7742 to 8367 due to missing data*.

### Measures

#### Oral reading fluency

Oral reading fluency was assessed with the *Dynamic Indicators of Basic Early Literacy* (DIBELS; Good and Kaminski, 1996) four times per year in September, December, February, and April from second through third grade. To measure ORF, students were presented with a passage of grade-level text and were asked to read for 1 min. Test administrators recorded the number of correct words read during a 1 min period. This procedure was conducted three times within the same observation, and the median score was recorded in words correct per minute for each wave of data. DIBELS ORF has been shown to have concurrent validity with the Test of Oral Reading Fluency (Children's Educational Services, 1987) and alternate-form reliabilities ranging from 0.89–0.96 when administered in second grade (Good et al., 2002).

#### Data analysis

First, we estimated the most theoretically representative model (Full model). The Full model was a four-level growth model accounting for time (Level 1) nested within students (Level 2), students cross-classified by second-and third-grade classrooms (Level 3), and classrooms nested in schools (Level 4). Conceptually, the Full model was most representative of the data as it included all theoretically important sources of contextual dependency, and thus serves as the reference model to which the other models will be compared. To confirm the Full model is the most appropriate model for the data, we conduct model comparisons based on model deviance statistics. The description of the Full model includes fixed and random effects. One way that Raudenbush and Bryk (2002) suggest interpreting the size of classroom or school random effects is by adding and subtracting the SD of a random effect to the average fixed effect of that parameter. For example, if the mean intercept was 100 and the school-level variance for the intercept was 25, then students in a classroom with an effect 1 SD above the mean would have a predicted intercept of 100 + √25 = 105. This could be compared to students in a classroom with an effect 1 SD below the mean, 100 − √25 = 95. Classroom and school effects were calculated using this method so that readers could make judgments about the practical significance of the variability of classrooms and schools.

To examine differences in fixed effects, random effects, and standard errors between growth models accounting for different levels of variance, we estimated four additional unconditional growth models with varying levels of data. All models were estimated using the lmer function (Bates and Maechler, 2009) in the R statistical program (R Development Core Team, 2005). The estimation method was specified to be restricted maximum likelihood because it is more computationally efficient than full maximum likelihood (Snijders and Bosker, 1999), and it takes the uncertainty of the fixed effects into account whereas full maximum likelihood does not (Raudenbush and Bryk, 2002). Also, an unstructured covariance matrix was specified to allow for the correlation between variances of the growth parameters at each level. The unstructured option places no restrictions on the relationships among variance parameters and is desirable when degrees of freedom are available and the exact structure of the data is unknown (Singer and Willett, 2003).

The four comparison models represent various model misspecifications. First, a two-level growth model accounting for time nested within students (Student model) was estimated because of the commonality of this type of model in educational research. The Student model ignores classroom and school effects altogether. Second, a three-level growth model accounting for time nested within students nested within schools (School model) was estimated as a possible anecdote to ignoring the clustered structure of the data. However, the School model ignores classroom effects. Then, a three-level growth model accounting for time nested within students nested within second-grade classrooms (Classroom model) was estimated as an alternate anecdote to ignoring higher-level variance. This model ignores both the cross-classification of students by third grade classrooms and the nesting of classrooms within schools. Fourth, a three-level growth model was estimated to account not only for time nested within students but also students cross-classified by second-grade classrooms and third-grade classrooms (Cross-Classified model). The Cross-Classified model ignores the nesting of classrooms within schools.

**Figure 1:**
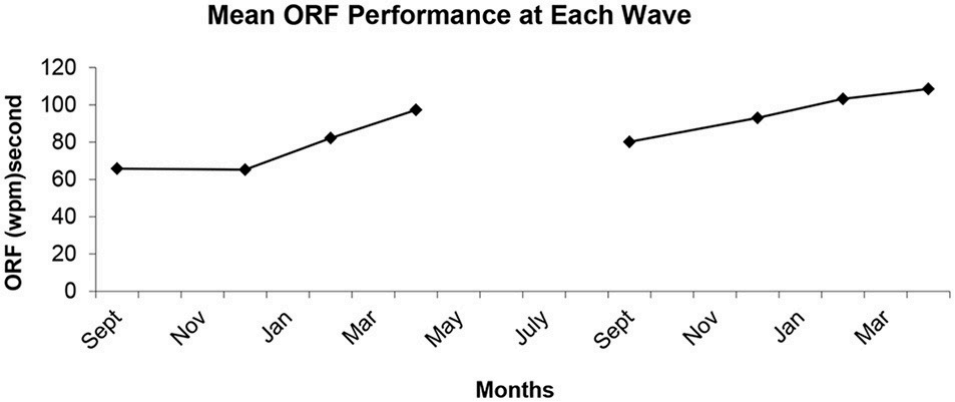
**Mean oral reading fluency levels at each wave of data collection across second and third grades**.

Singer and Willett (2003) recommend that the first step in modeling growth should be estimation of unconditional means models (no predictors included) to provide information about the proportion of variance lying at each level. Therefore, each of the models described in the previous section were estimated without the addition of growth parameters. Then, five growth models were estimated. Growth in ORF was estimated with a dummy-coded model such that the level of performance, the rate of growth at the end of the year, and the acceleration during the year were estimated independently for grades 2 and 3. Acceleration terms were included because the plotted mean ORF scores at each wave revealed a visible curve to the data in both grades (see Figure 1), the proportion of variance explained by the addition of the acceleration terms over simple growth terms was 24.36%, and the acceleration terms had high *t*-values in the final (Full) model (see **Table 4**).

Separate growth parameters for different time periods are useful for comparing the estimates and variability of those growth parameters. More traditional two-piece models like those presented in Raudenbush and Bryk (2002) could not be used with these data because of the summer gap between second and third grade. In a traditional two-piece model, growth or decline during the summer period would have to be included at the end of second grade or the beginning of third grade. Because students do not maintain the same rate of growth during academic year and summer periods, especially for lower socioeconomic populations (Cooper et al., 1996), combining the summer period with either second or third grade would have downwardly biased those estimates. Instead, we chose to use dummy coding so that the model would provide an intercept (end of grade 3), slope for end of the grade 3 year, and acceleration for grade 3, and deflections from each term for grade 2. Intercept and slope terms should be interpreted as year-end estimates because time was centered at the end of each grade. See Appendix A in Supplementary material for dummy-coded time variables. Al Otaiba et al. (2009) also used this type of coding scheme in modeling the second and third grade curvilinear growth trajectories of students for whom English was a second language.

Model equations as well as R syntax for the full model appear in Appendix B in Supplementary material. In all five models, the intercept, π_0,_ represents the ORF level at the end of third grade, π_1_ represents the instantaneous rate of growth at the end of third grade, π_2_ represents the acceleration of growth during third grade, π_3_ the difference in ORF level at the end of second grade from the level in third grade, π_4_ represents the difference in instantaneous rate of growth at the end of second grade from the rate of growth at the end of third grade, and π_5_ represents the difference in acceleration of second grade from that of third grade. To calculate the ORF level for the end of second grade, π_3_ was added to π_0_. Similarly, the rate of growth at the end of second grade was calculated by adding π_4_ to π_2_. Finally, the acceleration of growth in second grade was calculated by adding π_5_ to π_3._ Acceleration terms were not allowed to vary across units as the models would not converge without first fixing those terms. Crowe et al. (2009) also found it necessary to fix the random components of acceleration terms when modeling growth in ORF across grades 1–3. We used the most the theoretically representative model (Full Model) as the standard by which to judge the other models in terms of bias in the variance components and standard errors.

## Results

### Description of full model

Intraunit correlation coefficients (IUCC) for the unconditional means models are presented in the fifth column of Table 3. IUCCs for cross-classified models are equivalent to ICCs for nested models, and they represent the proportion of variance at a given level in relation to the total variance. For example, the equation for calculating the IUCC at the student level is:

(1) $$\begin{array}{l} \rho_{a}\;=\;\frac{\tau_{a}}{\tau_{a}\;+\;\tau_{b}+\;\tau_{c}\;+\;\tau_{d}\;+\;\sigma^{2}} \end{array}$$

where τ_*a*_ is the variance due to students, τ_*b*_ is the variance due to second-grade classrooms, τ_*c*_ is the variance due to third-grade classrooms, τ_*d*_ is the variance due to schools, and σ^2^ is the residual (or error) variance. IUCCs for the Full model revealed that the majority of the variance in the means lies between students as 54.4% of the variance was attributable to the student level. The proportion of variance at the second grade classroom, third grade classroom, and school levels was 5.6, 8.2, 2.5%, respectively. These estimates are similar to those found by Han (2008) who modeled growth in reading achievement of immigrant children across grades K-3. The school ICC was somewhat elevated (9%) compared to our study, most likely because classroom effects were not taken into account. Finally, the residual error variance accounted for 29.3% of the total variance. In growth models, the residual error variance represents the amount of within-person variation across time. Non-zero variance at this level indicates growth parameters should be added to explain the residual intra-individual variance not accounted for by the mean level of outcome (Singer and Willett, 2003). Thus, growth parameters were added to the models.

**Table 3:** **Proportion of variance explained at each level for unconditional means models (*N* = 8367)**.

		**Student model**	**School model**	**Classroom model**	**Cross-classified Model**	**Full model**
Student level		0.709	0.657	0.578	0.544	0.544
Classroom level						
	2nd grade			0.131	0.064	0.056
	3rd grade				0.098	0.082
School level			0.051			0.025
Error		0.291	0.292	0.290	0.294	0.293

*These estimates are also known as intraunit correlation coefficients (IUCCs)*.

The fixed estimates of the growth parameters along with their corresponding standard errors and *t*-values from the Full model are presented in the fifth column of Table 4. No *p*-values are reported because the lmer function provides model estimates based on (restricted) maximum likelihood methods, but *p*-values cannot be accurately calculated as likelihood methods do not assume symmetric parameter distributions (Bates, 2006a). According to the Full model, the average acceleration in third grade was −0.13 words per min (wpm) per month, indicating a deceleration of growth across third grade. The average instantaneous rate of growth at the end of third grade was 3.24 wpm, while the average ORF level at that time was 107.34 wpm. Second grade growth estimates were represented by dummy variables in the model, so interpretation requires adding the second grade estimate to the third grade estimate. After performing those calculations, the average acceleration in second grade was 0.95 wpm/month (−0.13 + 1.08, see Table 4). Average instantaneous rate of growth at the end of second grade was 11.36 wpm (3.24 + 8.12), and the average ORF level at the end of second grade was 96.44 wpm (107.34 – 10.90). The average ORF score in the spring of second grade was 86.71 wpm. The average instantaneous growth at the end of third grade was 4.25 wpm and the average ORF score in the spring of third grade was 104.00 wpm. The slower rate of growth at the end of third grade compared to second grade matches the findings of Deno et al. (2001) and Fuchs et al. (1993) that growth in word reading for both typical students and students in education tends to slow down as higher grades are reached.

**Table 4:** **Estimates of fixed parameters for growth models (*N* = 8367)**.

**Growth parameters**	**Student model**		**School model**		**Classroom model**		**Cross-classified model**		**Full model**	
	**Estimate (*SE*)**	***t***	**Estimate (*SE*)**	***t***	**Estimate (*SE*)**	***t***	**Estimate (*SE*)**	***t***	**Estimate (SE)**	***t***
Grade 2 deflection from grade 3 Acceleration	1.09 (0.01)	83.69	1.08 (0.01)	83.40	1.09 (0.01)	83.52	1.09 (0.01)	83.54	1.08 (0.01)	83.41
Grade 2 deflection from end of grade 3 slope	8.19 (0.10)	83.36	8.15 (0.12)	69.78	8.13 (0.10)	78.22	8.13 (0.10)	78.23	8.12 (0.12)	69.60
Grade 2 deflection from grade 3 intercept	−10.64 (0.18)	−59.57	−10.74 (0.42)	−25.37	−10.82 (0.26)	−42.40	−10.83 (0.26)	−42.42	−10.90 (0.42)	−25.88
Grade 3 acceleration	−0.13 (0.01)	−14.63	−0.13 (0.01)	−14.64	−0.13 (0.01)	−14.66	−0.13 (0.01)	−14.66	−0.13 (0.01)	−14.66
End of grade 3 slope	3.22 (0.07)	46.51	3.23 (0.09)	37.79	3.23 (0.07)	44.35	3.23 (0.07)	44.24	3.24 (0.09)	37.90
End of grade 3 intercept	108.94 (0.36)	299.39	108.83 (0.78)	138.94	107.53 (0.56)	191.46	107.14 (0.59)	181.95	107.34 (0.77)	139.53

Similar to the present study, Fuchs et al. (1993) also found that a notable portion of students had curvilinear growth patterns, and that there was a pattern for gradual deceleration over grades. It is difficult to compare growth rates presented in this study to those found in prior research studies (that employ linear growth models) because the presence of quadratic terms in our growth models alters the interpretation of slope terms. In linear growth models, a slope term represents the average rate of growth across a period of time. In quadratic growth models, however, the slope term represents the instantaneous rate of growth where time is equal to zero in the coding scheme. Therefore, slopes in our models represent the instantaneous rates of growth at the end of each grade, as that is where we chose to center time.

Because fixed growth parameters represent estimated averages, it is informative to examine the variability in those parameters. Table 5 presents IUCCs for each random growth parameter in each model. In growth models, IUCCs are calculated separately for each growth term (Raudenbush and Bryk, 2002). For example, the IUCC for the student-level variance in end-of-grade 3 intercept would be calculated from the variance estimates presented in Table 6: variance in end-of-grade 3 intercept at the student level/total variance in end-of-grade 3 intercept = 954.44/(954.44+71.02) = 0.93. Analogous to IUCCs for the Full unconditional means model, the Full growth model shows that the majority of variance in year-end slopes and intercepts is attributable to between-student differences. Between-school differences accounted for the next highest proportion of variance in the year-end slope parameters. Interestingly, second-grade classrooms accounted for more variance in end-of-third grade slopes than third-grade classrooms. Schools and second grade classrooms were equally influential on the difference between second and third grade ORF levels as they both accounted for approximately 22% of the total variance in that term.

**Table 5:** **Proportion of variance in growth parameters explained at each level (*N* = 8367)**.

**Level/Parameter**	**Student model**	**School model**	**Classroom model**	**Cross-classified model**	**Full model**
**STUDENT LEVEL**						
Grade 2 deflection from end of Grade 3 slope	1.00	0.74	0.55	0.55	0.56
Grade 2 deflection from Grade 3 intercept	1.00	0.80	0.72	0.72	0.72
End of Grade 3 slope	1.00	0.74	0.67	0.64	0.64
End of Grade 3 intercept	1.00	0.93	0.84	0.80	0.80
**CLASSROOM LEVEL**						
**2nd Grade**						
Grade 2 deflection from end of Grade 3 slope			0.45	0.45	0.22
Grade 2 deflection from Grade 3 intercept			0.28	0.28	0.09
End of Grade 3 slope			0.33	0.28	0.07
End of Grade 3 intercept			0.16	0.08	0.06
**3rd Grade**						
End of Grade 3 Slope				0.09	0.05
End of Grade 3 Intercept				0.12	0.09
**SCHOOL LEVEL**						
Grade 2 deflection from end of Grade 3 slope		0.26			0.22
Grade 2 deflection from Grade 3 intercept		0.20			0.18
End of Grade 3 slope		0.26			0.24
End of Grade 3 intercept		0.07			0.04

*Values also known as intraunit correlation coefficients (IUCC)*.

**Table 6:** **Estimates of random parameters for growth models (*N* = 8367)**.

**Level/Variance Components**	**Student model**		**School model**		**Classroom model**		**Cross-classified model**		**Full model**	
	**Variance**	***SD***	**Variance**	***SD***	**Variance**	***SD***	**Variance**	***SD***	**Variance**	***SD***
**STUDENT LEVEL**										
Grade 2 deflection from end of grade 3 slope	2.28	1.51	1.70	1.30	1.28	1.13	1.28	1.13	1.29	1.14
Grade 2 deflection from grade 3 intercept	108.31	10.41	86.60	9.31	78.27	8.85	78.32	8.85	78.64	8.87
End of grade 3 slope	1.43	1.19	1.05	1.03	0.96	0.98	0.88	0.94	0.90	0.95
End of grade 3 intercept	1028.64	32.07	954.44	30.89	865.27	29.42	809.33	28.45	814.19	28.53
**CLASSROOM LEVEL**										
**2nd Grade**										
Grade 2 deflection from end of grade 3 slope					1.04	1.02	1.04	1.02	0.52	0.72
Grade 2 deflection from grade 3 intercept					30.57	5.53	30.52	5.52	10.02	3.17
End of grade 3 slope					0.47	0.68	0.38	0.62	0.09	0.30
End of grade 3 intercept					168.75	12.99	84.37	9.19	63.31	7.96
**3rd Grade**										
End of grade 3 slope							0.12	0.34	0.07	0.26
End of grade 3 intercept							116.92	10.81	96.22	9.81
**SCHOOL LEVEL**										
Grade 2 deflection from end of grade 3 slope			0.59	0.77					0.50	0.70
Grade 2 deflection from grade 3 intercept			21.81	4.67					19.87	4.46
End of grade 3 slope			0.37	0.61					0.34	0.59
End of grade 3 intercept			71.02	8.43					42.34	6.51
**ERROR**	85.13	9.23	85.12	9.23	85.12	9.23	85.10	9.23	85.12	9.23
**GOODNESS-OF-FIT**										
Deviance	534383		532803		532699		532471		532033	
ΔDeviance	2350.00	^*^	770.63	^*^	666.39	^*^	437.97	^*^	–	
AIC	534446		532880		532779		532557		532136	
BIC	534601		533126		533025		532830		532500	

* *Indicates model has significantly worse fit compared to Full model. AIC = Akaike's information criterion (smaller is better). BIC = Bayesian information criterion (smaller is better)*.

Table 6 presents random effects for all models. For the same reason *p*-values are not calculated by the lmer function, standard errors for random effects are also not produced (Bates, 2006b). Results from the Full model indicate that only a trivial amount of variability was detected in end-of-third-grade slopes across all levels (τ = 1.40: 0.90 + 0.09 + 0.07 + 0.34), while slightly more variability existed in the difference between end-of-year second and third grade slopes (τ = 2.51: 1.29 + 0.52 + 0.70). Compared to the slope parameters, the year-end intercept parameters had much more variability. The variance in the difference between end of grade 2 and end of grade 3 intercepts was 108.53 (78.64 + 10.02 + 19.87), while it was 1016.06 (814.19 + 63.31 + 96.22 + 42.34) in end of grade 3 intercept. Al Otaiba et al. (2009), Han (2008), and Kieffer (2008) also found that slope parameters for reading growth had less variation than intercept parameters, even though their samples were comprised of language minority populations. Furthermore, Al Otaiba et al. found very little variation in acceleration parameters (τ = 0.30 and 0.20) across second and third grades.

Classroom and school effects were calculated using the random variance components results from the Full Model. These results are provided so that readers may judge the practical significance of the contextual effects. Results are presented in the order of second grade classrooms, third grade classrooms, and schools. According to the results, students had a predicted end-of-third-grade growth rate of 3.54 wpm (3.24 [from Table 3] + √0.09 [from Table 6]) when they were members of second-grade classrooms with an effect of one SD above the mean. For students in second-grade classrooms with an effect one SD below the mean, the predicted growth rate dropped to 2.94 wpm/month (3.24 [from Table 3] - √0.09 [from Table 6]). Also, students in second-grade classrooms with an effect of one SD above the mean were predicted to have an end of third-grade ORF level of 115.30 wpm, but were predicted to have an ORF level of 99.38 wpm if they were in a classroom with an effect of one SD below the mean. The differences in second and third grade slopes and intercepts were also affected by second-grade classroom membership. The difference in instantaneous rates of growth at the end of second grade compared to third grade was 8.84 wpm for students who were members of second-grade classrooms with an effect one SD above the mean, but the difference was 7.40 wpm in classrooms with an effect one SD below the mean. Finally, the difference in end of second-grade ORF levels compared to end of third-grade ORF levels was −4.56 wpm for students in second-grade classrooms with an effect one SD above the mean compared to −14.07 wpm for students in second-grade classrooms with an effect one SD below the mean. Because growth in word-reading tends to slow down over time, a wider gap in the negative direction between ORF levels in second and third grade is not a desirable outcome.

For third-grade classrooms with an effect one SD above the mean, students were predicted to have a 0.53 wpm advantage in instantaneous third-grade growth rate over students in third-grade classrooms with an effect one SD below the mean. Similarly for end of third-grade intercepts, there was a predicted 6.26 wpm advantage for students in third-grade classrooms with an effect one SD above the mean as opposed those in classrooms one SD below the mean.

If students had two consecutive above-average effect classrooms, their predicted end of third-grade ORF level 125.11 wpm compared to 89.57 wpm for the unfortunate students in two consecutive classrooms having an effect one SD below the mean. Although no classroom-level variables were used to predict the between-classroom variation, our models suggest that investigation could be a worthwhile endeavor.

It terms of school effects, students attending schools with an effect of one SD above the mean had 1.35 wpm higher predicted instantaneous third-grade slopes than students attending schools with an effect of one SD below the mean. The difference between reading levels of students attending a school with an effect one SD above the mean and students attending a school with an effect on SD below the mean was 9.06 wpm at the end of third grade. School effects on the difference in second and third grade growth estimates were not as dramatic. In a school with an effect one SD above the mean, students were predicted to have a difference in second to third grade instantaneous slopes of 8.82 wpm; students in schools with an effect one SD below the mean were predicted to have a 7.28 wpm difference in instantaneous slopes. However, there was a noteworthy school effect on ORF levels. The difference in ORF level at the end of second grade compared to the end of third grade was predicted to be −6.37 wpm for students in schools with an effect one SD above the mean, while it was only predicted to be −12.94 wpm for students in schools with an effect on SD below the mean. Because the appropriateness of significance tests for random effects been questioned (Bates, 2006b, July 15), the calculations in this section should allow researchers to make their own judgments about whether classroom and school effects have practical significance.

### Model comparisons

The Full model serves as the standard to which all other models are compared primarily because it is the best theoretical representation of the data as it includes variance components for students, classrooms, and schools. Research has shown that characteristics of all three have some influence on student word-reading. In addition, the Full model produced the best model fit as measured by the χ^2^ test of deviance. When comparing the deviance of the Full model to the deviance of every other model, the significant χ^2^ revealed that the more constrained models produced significantly worse fit. Furthermore, the Full model has the lowest Akaike's information criterion (AIC) and Bayesian information criterion (BIC) compared to the other models. See Table 6 for model fit statistics.

#### Unconditional means models

In the model comparison section, we explore the answer to our main research question regarding the consequences of omitting classroom and school effects on cross-year growth models. IUCCs for each model are presented in Table 5. Compared to the Full model and in accordance with results from Moerbeek (2004), the variance of the lowest level (Student level) was the most overestimated in cases where the intermediate level (Classroom level) was omitted (i.e., Student and School models). These two models overestimated the proportion of variance attributable to the student level by 23.20 and 17.21%, respectively. One consequence of omitting higher levels of variance is overestimating variance at the lowest level, which could lead to inflated Type I errors. It also gives researchers the perception that there is a greater magnitude of differences between the lowest level units than actually exists, which may cause researchers to limit, or at least focus, research questions to only the lowest level.

Models where the highest level was omitted (Classroom and Cross-Classified models) had student-level proportion of variance estimates closer to the Full model. In fact, the proportion of variance at the student level was exactly the same for the Cross-Classified model and the Full model (0.54). It appears, therefore, that accounting for classroom variance with cross-classified random effects (Cross-Classified model) produced more accurate student-level variance estimates than simply accounting for students' second grade classroom membership (Classroom model). However, while the Cross-Classified model produced accurate student-level estimates, the classroom IUCCs were somewhat overestimated compared with the Full model. Model IUCCs suggest that variance may be misattributed when any level is omitted. These results will be compared to those of the growth models.

#### Growth models

Fixed and random effects for the Student model, the School model, the Classroom model, and the Cross-Classified model are displayed in the first four columns of Table 4. The fixed effects for the five growth models were quite similar across models. The deceleration in third grade was exactly the same across models at −0.13 wpm/month. At the end of third grade, the average instantaneous growth at the end of third grade ranged from 3.22 to 3.24 wpm, and the average ORF score ranged from 107.14 to 108.94 wpm. The difference in acceleration at the end of second grade compared to third grade had a very narrow range from 1.08 to 1.09 wpm/month. The range of differences in instantaneous rates of growth between the grades was 8.12–8.19 wpm, and the range of difference in end-of-year intercepts was −10.64 to −10.90 wpm. The variance components of these parameters, however, differed substantially across models.

The IUCC indicates the proportion of total variance between units at a given level. In growth parameters, IUCCs can be calculated separately for each growth term to indicate what proportion of variance in the intercept or slope is attributed to each level (Raudenbush and Bryk, 2002). It is calculated by dividing the variance of a particular parameter at a particular level by the total variance of that parameter. Table 6 contains IUCCs, and it reveals that variance attributed to the highest level included in each model was overestimated compared to the Full model. This result is similar to findings in both nested (Opdenakker and Van Damme, 2000; Hutchison and Healy, 2001; Tranmer and Steele, 2001; Moerbeek, 2004) and cross-classified data (Luo and Kwok, 2009). Each model will be compared to the Full model in the following sections.

#### Student model

The student model only accounted for time nested within students. The differences between this model and the Full model are substantial. Inclusion of cross-classified and nested levels in the Full model reduced the student-level variance by 36.71% in instantaneous third grade growth, 20.85% in third grade intercept, 43.52% in second grade deflection from third grade instantaneous growth, and 27.39% in the second grade deflection from third grade intercept. These results are worrisome as they suggest that the two-level Student model overestimated the amount of variance due to between-student differences by misattributing higher level (e.g., classrooms and schools) variance to the student level. In addition to inflated variances, the Student model produced deflated standard errors for the fixed parameters compared to the Full model. Compared to the Full model, standard errors for 4 of the 6 the fixed effects in the Student model were deflated: 16.67% deflation of the second grade deflection from third grade instantaneous growth SE, 57.14% deflation of the second grade deflection from third grade intercept SE, 22.22% deflation of the third grade instantaneous growth SE, and 53.25% deflation of the third grade intercept SE.

#### School model

The School model also appears to have overestimated the variance at the student level. The amount of variance attributed to between-student differences in the School model was reduced by adding classroom memberships in the Full model by 9.19–24.16% depending on the parameter. Similar to the Student model, the School model overestimated the variance due to between-student differences. This model also inflated the variance due to between-school compared to the Full model. This was expected from Moerbeek's (2004) results. The School model had unbiased standard errors. The most likely explanation for this finding is the relatively little variance in growth terms accounted for by classroom memberships (in the Full model). Though the omission of classroom effects did not affect the standard errors, bias in variance estimates was observed when classroom memberships were not included.

#### Classroom model

Results from the Classroom model (student and second-grade classroom levels only) were in agreement with Moerbeek's (2004) and Luo and Kwok's (2009) findings. The amount of bias in student-level variance components was minimal, but there was overestimation of variance at the classroom level (the remaining crossed factor). Compared to the Full model, the Classroom model had inflated variance components for each parameter at the classroom level ranging from 201 to 504% inflation depending on the growth term. The implication of overestimating variance due to second grade classroom membership is that school effects are attributed to between-classroom differences when school membership is not taken into account. In addition to inflated variances, the Classroom model produced deflated standard errors for some of the growth parameters compared to the Full model. Compared to the Full model, standard errors for 4 of the six fixed effects in the Classroom model were deflated: 16.67% deflation of the second grade deflection from third grade instantaneous growth SE, 38.10% deflation of the second grade deflection from third grade intercept SE, 22.22% deflation of the third grade instantaneous growth SE, and 27.27% deflation of the third grade intercept SE.

#### Cross-classified model

As in the Classroom model above and in accordance with prior research (Moerbeek, 2004; Luo and Kwok, 2009), the student variances in the Cross-Classified model looked fairly similar to those in the Full model. The proportions of variance of each growth parameter at the student-level were nearly exactly the same between the two models. Furthermore, the amount of variance only differed by 0.01–4.86 across the parameters. This is the expected result of lower-level estimates when the highest level of variance is ignored. Also expected was that both second and third grade classroom variance estimates were inflated compared to the Full model. The school variance that was ignored in this model was absorbed somewhat by both crossed classroom factors. As in the other models, the Cross-Classified model produced deflated standard errors for several of the growth parameters compared to the Full model: 16.67% deflation of the second grade deflection from third grade instantaneous growth SE, 38.10% deflation of the second grade deflection from third grade intercept SE, 22.22% deflation of the third grade instantaneous growth SE, and 23.38% deflation of the third grade intercept SE.

## Discussion

In modeling passage ORF growth in second and third grade, we found it necessary to include not only between-student differences but also the differences between classrooms and schools so that variance was partitioned correctly such that model parameters and their standard errors were unbiased. Other authors have also stressed the importance of including all necessary higher levels of influence in multilevel models (Opdenakker and Van Damme, 2000; Hutchison and Healy, 2001; Tranmer and Steele, 2001; Raudenbush and Bryk, 2002; Moerbeek et al., 2003; Moerbeek, 2004; Meyers and Beretvas, 2006; Luo and Kwok, 2009). From our results, we conclude that classrooms and schools affect student level of performance and growth above and beyond the students' own characteristics. This is not a surprising finding; however, the illustration of the statistical consequences of not including higher levels should provide the necessary evidence that researchers need to include such effects in their models.

The Full model included both the cross-classified effects of classrooms and the nested effects of schools on student growth in ORF, and thus we consider it the best theoretical representation of these data. When other models were compared to the Full model, conclusions about the adequacy of the models depend on objective. If the purpose of modeling growth is simply to describe growth using fixed effects, simple two-level models (time nested within student) may be adequate as we found that estimates of the growth parameters were similar across models. In prior studies, the effects on fixed parameter estimates have been generally unaffected in the case of balanced designs (Moerbeek et al., 2003; Moerbeek, 2004; Meyers and Beretvas, 2006) but biased in unbalanced designs (Moerbeek et al., 2003; Moerbeek, 2004; Luo and Kwok, 2009). However, in longitudinal designs, fixed effects estimates were found to be unbiased in unbalanced designs, too, as long as the number of observations (level 1) were the same for each level 2 unit (Ramirez et al., 1991; Luo and Kwok, 2009). Describing growth is common in the field of education as average growth or progress for a student is frequently reviewed to help describe how student are responding to a particular instructional setting. In this situation, description rather than prediction is the primary goal, and two-level models appear adequate.

Many studies of reading development are not simply focused on the strict modeling of outcome performance, and are rather more interested in explaining why individuals differ in their performance. In such types of research, the results from a two-level model becomes invalid when there are important clustering components in the data. The likelihood of clustering in student data is great because, in reality, students are not randomly assigned to classrooms or schools. We found this to be true of our sample. When the variance components of the Student model were compared to the Full model, the estimate of variability attributed to students was too high and standard errors for fixed effects were too low. An implication of the shifting variances is that when once attempts to explain the variance with predictions, the amount and interpretation of variance explained at each level will vary sharply based on the selected model. In the student model, where 71% of the base variance in ORF was due to between-student differences, the implication of modeling covariates at the student level is greater than in the cross-classified and full models (54%). If a predictor, such as socioeconomic status (SES), explained 20% of the variance in ORF scores, there is a very different interpretation about the importance of SES when it's modeled in a student vs. cross-classified model. As the former model suggests that nearly three-quarters of the variance is due to students versus only half in the latter model, the implications of SES as a predictor might vary depending on how the data design is structured.

Similar conclusions were drawn with regard to the Classroom and Cross-Classified models. Variances at the highest level in those models were inflated, and the standard errors of the fixed effects were too small compared to the Full model. Again, these results of biased variance components and standard errors are consistent with those reported by other researchers, and especially true for unbalanced designs (Raudenbush and Bryk, 2002; Moerbeek et al., 2003; Moerbeek, 2004; Meyers and Beretvas, 2006; Luo and Kwok, 2009). While the School model produced unbiased standard errors, it was not the best representation of the data because the variance associated with second- and third-grade classrooms was included in between-school differences. Another consequence of ignoring higher levels of nesting is the inability to explain variance in terms of higher-level predictors (see for example, Raudenbush and Bryk, 2002), such as class-wide achievement or school composition. Although our recommendation regarding the necessity of including higher levels is consistent with the literature, our results concerning the precise effects on fixed estimates, variance components, and standard errors were difficult to compare with other studies because of the disparity in the type of growth parameters estimated in the current study and those found in prior studies.

### Limitations

Our study had two major limitations. First, we limited our sample to students who remained in the same classroom within a grade and the same school between grades. Some students left the study completely, while other simply changed classrooms or schools. Multilevel modeling requires students to be identified in one unit at each level, thus assigning students to more than one classroom (within a grade) or school was not feasible. By excluding movers, our sample may be biased and our results may only generalize to students who do not often change classrooms or schools.

Another limitation is that schools represented in this study were part of the *Reading First* program, which may have resulted in less variability between schools in terms of student reading achievement than what might be expected between schools in general. If any bias was associated with the population of schools in our study, it would be that school and classroom effects were underestimated, making results conservative with respect to the estimates of variability between schools and classrooms. In light of the study limitations and in order to extend the recommendations we have made, models used in this study should be replicated with more variable samples of schools in different grades using different outcomes.

### Recommendations

Despite the limitations of this study, we can make two recommendations based on our results. Because variance in cross-year ORF growth parameters was attributed to students, classrooms, and schools and because variance was partitioned appropriately when all were included in the model (Full model), we recommend including all levels of potential variation when modeling or predicting variance in ORF passage growth. One exception is when the sole purpose of modeling is to describe growth. In that case, including or omitting higher levels does not appear to affect the fixed effects coefficients. The other exception to our recommendation is when the following three conditions are met (on outcomes other than ORF): the amount of variance at any given level is theoretically and statistically nil, no predictors at that level are of interest, and the model fit statistics suggest the model without the level fit the data better than one with the level. In that case, the level is not necessary to include (Raudenbush and Bryk, 2002).

The second recommendation is that all possible levels of influence should be taken into account when conducting power analyses for future studies. Konstantopoulos (2008) determined through a simulation study that the number of schools (the highest level units) in a study had the greatest impact on power, followed by the number of classrooms, and then students. Snijders and Bosker (1999) foreshadowed this recommendation when they stated, “the sample size at the highest level is usually the most restrictive element in the design” (p. 140). In order to obtain sufficient power, it appears classrooms and schools should be included.

## Author contributions

JG, YP, DC, and CS contributed to the analysis, design, and write-up of the study.

## Funding

The research reported here was supported by the Institute of Education Sciences, U.S. Department of Education, in part by Grant #R324G0600036 and by Grant #R305B040110 to Vanderbilt University and Grant # R305A120147 to Florida State University and by the National Institutes of Health P50HD052120 to Florida State University. The opinions expressed are those of the authors and do not represent views of the U.S. Department of Education nor the National Institutes of Health.

### Conflict of interest statement

The authors declare that the research was conducted in the absence of any commercial or financial relationships that could be construed as a potential conflict of interest.

## Footnotes

^1^Lower-level units that are crossed by higher-level units can be considered nested in specific unit-by-unit combinations. However, combining the higher-level units in this way precludes estimating the unique variance associated with each.

^2^Act of 2001, Pub. L. No. 107-110, § 115, Stat. 1425.
