# Supplementary material for: Consequences of Misspecifying Levels of Variance in Cross-Classified Longitudinal Data Structures
Source: Front Psychol. 2016 May 18;7:695. doi: 10.3389/fpsyg.2016.00695 (PMC4870234; doi:10.3389/fpsyg.2016.00695)
Supplement: Supplementary file 1 [file DataSheet1.docx]

Appendix A

|  | Sept. Grade 2 | Dec. Grade 2 | Feb. Grade 2 | April Grade 2 | Sept. Grade 3 | Dec. Grade 3 | Feb. Grade 3 | April Grade 3 |
| --- | --- | --- | --- | --- | --- | --- | --- | --- |
| *π*_1_, *Months*  (Grade 3 slope) | -7 | -4 | -2 | 0 | -7 | -4 | -2 | 0 |
|  |  |  |  |  |  |  |  |  |
| *π*_2_, *Months*Months*  (Grade 3 acceleration) | 49 | 16 | 4 | 0 | 49 | 16 | 4 | 0 |
|  |  |  |  |  |  |  |  |  |
| *π*_3_, *Grade2*  (Grade 2 deflection from Grade 3 intercept) | 1 | 1 | 1 | 1 | 0 | 0 | 0 | 0 |
|  |  |  |  |  |  |  |  |  |
| *π*_4_,*Months*Grade2*  (Grade 2 deflection from Grade 3 slope) | -7 | -4 | -2 | 0 | 0 | 0 | 0 | 0 |
|  |  |  |  |  |  |  |  |  |
| *π*_5_, *Months*Months*Grade2*  (Grade 2 deflection from Grade 3 acceleration) | 49 | 16 | 4 | 0 | 0 | 0 | 0 | 0 |

Coding of time for two-piece growth model with time centered at the April testing wave of each grade. Negative values for the slope term reflect the number of months prior to April testing.

Appendix B

*Student model*:

Level 1 (Time): *Y_ti_* = *π*_0_*_i_* + *π*_1_*_i_Months_ti_* + *π*_2_*_i_Months_ti_*Months_ti_* + *π*_3_*_i_Grade2_ti_* + *π*_4_*_i_Months_ti_*Grade2_ti_* + *π*_5_*_i_Months_ti_*Months_ti_*Grade2_ti_+ e_ti_*

Level 2 (Student): *π*_0_*_i_* = *β*_00_ + *r*_0_*_i_*

*π*_1_*_i_* = *β*_10_ + *r*_1_*_i_*

*π*_2_*_i_* = *β*_20_

*π*_3_*_i_* = *β*_30_ + *r*_3_*_i_*

*π*_4_*_i_* = *β*_40_ + *r*_4_*_i_*

*π*_5_*_i_* = *β*_50_

*School model*:

Level 1 (Time): *Y_tik_* = *π*_0_*_ik_* + *π*_1_*_i_Months_tik_* + *π*_2_*_i_Months_tik_*Months_tik_* + *π*_3_*_i_Grade2_tik_* + *π*_4_*_i_Months_tik_*Grade2_tik_* + *π*_5_*_i_Months_tik_*Months_tik_*Grade2_tik_* + *e_tik_*

Level 2 (Student): *π*_0_*_ik_* = *β*_00_ + *r*_0_*_ik_*

*π*_1_*_ik_* = *β*_10_ + *r*_1_*_ik_*

*π*_2_*_ik_* = *β*_20_

*π*_3_*_ik_* = *β*_30_ + *r*_3_*_ik_*

*π*_4_*_ik_* = *β*_40_ + *r*_4_*_ik_*

*π*_5_*_ik_* = *β*_50_

Level 3 (School): *β*_00_*_k_* = *γ*_000_ + *u*_00_*_k_*

*β*_10_*_k_* = *γ*_100_ + *u*_10_*_k_*

*β*_20_*_k_* = *γ*_200_

*β*_30_*_k_* = *γ*_300_ + *u*_30_*_k_*

*β*_40_*_k_* = *γ*_400_ + *u*_40_*_k_*

*β*_50_*_k_* = *γ*_500_

*Classroom model*:

Level 1 (Time): *Y_tij_* = *π*_0_*_ij_* + *π*_1_*_i_Months_tij_* + *π*_2_*_i_Months_tij_*Months_tij_* + *π*_3_*_i_Grade2_tij_* + *π*_4_*_i_Months_tij_*Grade2_tij_* + *π*_5_*_i_Months_tij_*Months_tij_*Grade2_tij_* + *e_tij_*

Level 2 (Student): *π*_0_*_ij_* = *β*_00_*_j_* + *r*_0_*_ij_*

*π*_1_*_ij_* = *β*_10_*_j_* + *r*_1_*_ij_*

*π*_2_*_ij_* = *β*_20_*_j_*

*π*_3_*_ij_* = *β*_30_*_j_* + *r*_3_*_ij_*

*π*_4_*_ij_* = *β*_40_*_j_* + *r*_4_*_ij_*

*π*_5_*_ij_* = *β*_50_*_j_*

Level 3 (2^nd^ grade classroom): *β*_00_*_j_* = *γ*_000_ + *u*_00_*_j_*

*β*_10_*_j_* = *γ*_100_ + *u*_10_*_j_*

*β*_20_*_j_* = *γ*_200_

*β*_30_*_j_* = *γ*_300_ + *u*_30_*_j_*

*β*_40_*_j_* = *γ*_400_ + *u*_40_*_j_*

*β*_50_*_j_* = *γ*_500_

*Cross-classified model*:

Level 1 (Time):

*Y_ti(j_*_1_*_j_*_2_*_)_* = *π*_0_*_i(j_*_1_*_j_*_2_*_)_* + *π*_1_*_i(j_*_1_*_j_*_2_*_)_Months_ti(j_*_1_*_j_*_2_*_)_* + *π*_2_*_i(j_*_1_*_j_*_2_*_)_Months_ti(j_*_1_*_j_*_2_*_)*_ Months_ti(j_*_1_*_j_*_2_*_)_* + *π*_3_*_i(j_*_1_*_)_Grade2_ti(j_*_1_*_)_* + *π*_4_*_i(j_*_1_*_)_Months_ti(j_*_1_*_)_*Grade2_ti(j_*_1_*_)_* + *π*_5_*_i(j_*_1_*_)_Months_ti(j_*_1_*_)_*Months _ti(j_*_1_*_)_*Grade2_ti(j_*_1_*_)_* + *e_ti(j_*_1_*_j_*_2_*_)_*

Level 2 (Student): *π*_0_*_i(j1j_*_2_*_)_* = *β*_00(_*_j_*_1_*_j_*_2_*_)_* + *r*_0_*_i(j_*_1_*_j_*_2_*_)_*

*π*_1_*_i(j1j_*_2_*_)_* = *β*_10(_*_j_*_1_*_j_*_2_*_)_* + *r*_1_*_i(j_*_1_*_j_*_2_*_)_*

*π*_2_*_i(j_*_1_*_j_*_2_*_)_* = *β*_20(_*_j_*_1_*_j_*_2_*_)_*

*π*_3_*_i(j_*_1_*_)_* = *β*_30(_*_j_*_1_*_)_* + *r*_3_*_i(j_*_1)_

*π*_4_*_i(j_*_1_*_)_* = *β*_40(_*_j_*_1_*_)_* + *r*_4_*_i(j_*_1)_

*π*_5_*_i(j_*_1_*_)_* = *β*_50(_*_j_*_1_*_)_*

Level 3 (2^nd^ & 3^rd^ grade classroom): *β*_00(_*_j_*_1_*_j_*_2_*_)_* = *γ*_000_ + *b*_00_*_j_*_1_ + *c*_00_*_j_*_2_

*β*_10(_*_j_*_1_*_j_*_2_*_)_* = *γ*_100_ + *b*_10_*_j_*_1_ + *c*_10_*_j_*_2_

*β*_20(_*_j_*_1_*_j_*_2_*_)_* = *γ*_200_

*β*_30(_*_j_*_1_*_)_* = *γ*_300_ + *b*_30_*_j_*_1_

*β*_40(_*_j_*_1_*_)_* = *γ*_400_ + *b*_40_*_j_*_1_

*β*_50(_*_j_*_1_*_)_* = *γ*_500_

*Full model*:

Level 1 (Time):

*Y_ti(j_*_1_*_j_*_2_*_)k_* = *π*_0_*_i(j_*_1_*_j_*_2_*_)k_* + *π*_1_*_i(j_*_1_*_j_*_2_*_)k_Months_ti(j_*_1_*_j_*_2_*_)k_*  + *π*_2_*_i(j_*_1_*_j_*_2_*_)k_Months_ti(j_*_1_*_j_*_2_*_)k_*Months_ti(j_*_1_*_j_*_2_*_)k_*  + *π*_3_*_i(j_*_1_*_)k_Grade2_ti(j_*_1_*_)k_* + *π*_4_*_i(j_*_1_*_)k_Months*Grade2_ti(j_*_1_*_)k_* + *π*_5_*_i(j_*_1_*_)k_Months _ti(j_*_1_*_)k_*Months_ti(j_*_1_*_)k_*Grade2_ti(j_*_1_*_)k_* + *e_ti(j_*_1_*_j_*_2_*_)k_*

Level 2 (Student): *π*_0_*_i(j_*_1_*_j_*_2_*_)k_* = *β*_00(_*_j_*_2_*_)k_* + *r*_0_*_i(j_*_1_*_j_*_2_*_)k_*

*π*_1_*_i(j_*_1_*_j_*_2_*_)k_* = *β*_10(_*_j_*_2_*_)k_* + *r*_1_*_i(j_*_1_*_j_*_2_*_)k_*

*π*_2_*_i(j_*_1_*_j_*_2_*_)k_* = *β*_20(_*_j_*_1_*_j_*_2_*_)k_*

*π*_3_*_i(j_*_1_*_)k_* = *β*_30(_*_j_*_1_*_)k_* + *r*_3_*_i(j_*_1_*_)k_*

*π*_4_*_i(j_*_1_*_)k_* = *β*_40(_*_j_*_1_*_)k_* + *r*_4_*_i(j_*_1_*_)k_*

*π*_5_*_i(j_*_1_*_)k_* = *β*_50(_*_j_*_1_*_)k_*

Level 3 (2^nd^ & 3^rd^ grade classroom): *β*_00(_*_j_*_1_*_j_*_2_*_)k_* = *γ*_000k_+ *b*_00_*_j_*_1_*_k_* + *c*_00_*_j_*_2_*_k_*

*β*_10(_*_j_*_1_*_j_*_2_*_)k_* = *γ*_100k_+ *b*_10_*_j_*_1_*_k_* + *c*_10_*_j_*_2_*_k_*

*β*_20(_*_j_*_1_*_j_*_2_*_)k_* = *γ*_200k_

*β*_30(_*_j_*_1_*_)k_* = *γ*_300k_+ *b*_30_*_j_*_1_*_k_*

*β*_40(_*_j_*_1_*_)k_* = *γ*_400k_+ *b*_40_*_j_*_1_*_k_*

*β*_50(_*_j_*_1_*_)k_* = *γ*_500(_*_j_*_1_*_)_*_k_

Level 4 (School): *γ*_000_*_k_* = *θ*_0000_ + *u*_00_*_k_*

*γ*_100_*_k_* = *θ*_1000_ + *u*_10_*_k_*

*γ*_200_*_k_* = *θ*_2000_

*γ*_300_*_k_* = *θ*_3000_ + *u*_30_*_k_*

*γ*_400_*_k_* = *θ*_4000_ + *u*_40_*_k_*

*γ*_500_*_k_* = *θ*_5000_

where *π*’s represent fixed parameters at level 1; *β*’s represent fixed parameters at level 2; *γ*’s represent fixed parameters at level 3; and *θ*’s represent the fixed parameters at level 4; *e* represents the random parameter at level 1; *r*’s represent random parameters at level 2; *b*’s and *c*’s represent random parameters at level 3 for second and third grade classrooms, respectively; and *u*’s represent random parameters at level 4.

R syntax for the full model: (fullmodel<-lmer(orf ~ 1 + gr2 + grade2a + grade2asq + months3 + months3sq + (1 + grade2a + gr2 + months3 | studentID) + (1 + grade2a + months3 + gr2 | grade2teacherID) + (1 + months3 | grade3teacherID) + (1 + grade2a + months3 + gr2 | schoolID), mydata, verbose=TRUE))
